# Corporate Social Responsibility Activities and Green Innovation Performance in Organizations: Do Managerial Environmental Concerns and Green Absorptive Capacity Matter?

**DOI:** 10.3389/fpsyg.2022.938682

**Published:** 2022-07-07

**Authors:** Xiaoping Mo, Francis Boadu, Yunqing Liu, Zhen Chen, Adwoa Serwaa Ofori

**Affiliations:** ^1^School of Business Administration, Chongqing Technology and Business University, Chongqing, China; ^2^Faculty of Entrepreneurship and Enterprise Development, Kumasi Technical University, Kumasi, Ghana; ^3^Institute of Finance and Public Administration, Anhui University of Finance and Economics, Bengbu, China; ^4^Dazhou Central Hospital, Dazhou, China; ^5^Registry, Kumasi Technical University, Kumasi, Ghana

**Keywords:** corporate social responsibility activities, managerial environmental concern, green absorptive capacity, green innovation performance, moderated mediating

## Abstract

From the environmental sustainability perspective, scholars have considered corporate social responsibility activities as an essential mechanism for enhancing enterprise performance and innovation outcomes. However, how and under what conditions corporate social responsibility activities influence green innovation performance in emerging economies is still unclear. From the perspective of the theory of planned behavior, we construct a theoretical model to assess how corporate social responsibility activities affect enterprises’ green innovation performance. Explicitly, we investigate the mediating and moderating effects of managerial environmental concern and green absorptive capacity on the relationship between corporate social responsibility activities and enterprises’ green innovation performance. This research relies on a sample of 358 enterprises from the manufacturing and service sectors in China, and uses regression analysis and bootstrap to test the hypotheses proposed. The empirical results demonstrate that (1) corporate social responsibility activities positively enhance enterprises’ green innovation performance; (2) corporate social responsibility activities have a positive influence on managerial environmental concern; (3) managerial environmental concern has a mediating role between corporate social responsibility activities and green innovation performance; (4) managerial environmental concern has a powerful influence on green innovation performance; (5) green absorptive capacity positively moderates the association between managerial environmental concern and green innovation performance. This research work proposes that managerial environmental concern and green absorptive capacity play a mediating and moderating function on the linkage amongst corporate social responsibility activities and green innovation performance.

## Introduction

Green innovation has become a critical driving element in the twenty-first century for firm growth, development, and survival. The concept has received attention from academicians, practitioners, and civil service organizations due to its direct impact on the environment. For the past few years, the public and other stakeholders have gradually become aware of green innovation’s significance to society and the impact of enterprise activities on the environment (Wong, 2013; Song et al., 2021). However, compared with advanced market enterprises, emerging market enterprises face superior challenges to strike an equilibrium sandwiched between development and the environment. For instance, the People’s Republic of China is faced with ecological and environmental menace, which threatens and undermines the sustainable development of enterprises due to its rapid industrialization drive (Ebenstein, 2012). For enterprises to survive in today’s turbulent market landscape, scholars have recognized corporate social responsibility (CSR) activities as an essential missile for improving the evolving sustainability practices (Hsu, 2012). CSR activities are the sum of the voluntary actions taken by an enterprise to meet the economic, ethical, social, and environmental anticipations of individuals and the community (Shum and Yam, 2011). In major global enterprises, CSR activities provide corrective measures to mitigate environmental menace toward enterprise performance. Previous CSR scholarships have pointed out that top-level executives play a leadership role in executing enterprises’ CSR activities.

Admittedly, authors have recognized the function of CSR in the chain as a significant driver of achieving the enterprise’s sustainable and superior performance (Saeidi et al., 2015). It lays a foundation for enterprises to implement cleaner production technology and eco-friendly production mode. Previous scholarships have affirmed the direct effect of the CSR dimension on enterprise performance and innovation capability. In contrast, others have proved an indirect relationship between CSR dimension and enterprise performance and innovation capability. However, how managers’ perceived behaviors influence CSR activities’ toward the green innovation performance (GIP) of enterprises has not received adequate empirical consideration, especially in emerging economies (Ji et al., 2019; Ji and Miao, 2020). The reasons for choosing green innovation performance as a research variable in the present scholarship can be addressed in three ways. First, the GIP can effectually mirror an enterprise’s efforts in green innovation activities (Xue et al., 2019; Khalil and Nimmanunta, 2022). Second, GIP assessment is a more objective tactic to measure an enterprise’s sustainable performance. Finally, current trends in GIP research (Padilla-Lozano and Collazzo, 2022) have led to a renewed interest in the function of green innovation management in the emerging markets (Ji et al., 2019; Ji and Miao, 2020). In addition, most studies focus on how the inner and outer factors such as stakeholders (Chang, 2016; Hadj, 2020), corporate governance (Yi et al., 2012; Honoré et al., 2015), social value (Broadstock et al., 2020; Liu et al., 2021), and evaluation system (Borger and Kruglianskas, 2006; Yang et al., 2016) affect CSR activities and innovation performance.

The present study intends to address this gap in the extant work by scrutinizing how CSR activities affect GIP *via* managerial environmental concern (MEC) (i.e., mediator) and green absorptive capacity (GAC) (i.e., moderator) in emerging economies, especially, China. The Chinese environment is an ideal economy to assess the research model or variables. From a global perspective, the country is ranked as the largest emerging economy and has many common characteristics with other emerging economies. Over the past few years, the country has grown considerably without a threat to its rapid industrialization drive (Ebenstein, 2012). Nevertheless, in recent years, the policymakers have devoted an inordinate connotation to eco-friendly matters and implemented the concept of a green revolution in innumerable enterprises to drive sustainability (Zhou et al., 2018). Besides, the Chinese government has roll-out several policies to uphold enterprises’ sustainable growth (Chen et al., 2017). Certainly, the environment provides a fertile ground to scrutinize the association between CSR activities, GIP, MEC, and GAC. The current study is novel, enthralling, and crucial that will considerably contribute to green innovation potentials in the existing literature.

First, MEC in a corporate decision, operational activity, and strategic blueprint has gradually become one of the important subjects in environmental management (Gholami et al., 2013). MEC describes the top management’s commitment level to the enterprise’s environmental matters (Xue et al., 2019). As top executives are the key decision-makers in enterprises, their commitment to environmental sustainability has a robust influence on the enterprise’s strategies and activities associated with green practices. Thus, executives’ commitment affects the rapidity at which enterprises initiate ecological activities (Song et al., 2021). Some scholarships shed light on the importance of the managers’ environmental values and concerns regarding environmental issues (Boiral et al., 2018), as well as their decisions to integrate environmental strategies into their business models (Aragon-Correa and Sharma, 2003). Boiral et al. (2018) assert that resource allocation for environmental management practice relies on top management commitment. For top executives, their self-environmental perception could impact the daily operations of firms. Predominantly in emerging economies where regulatory structures may not be fully developed, managerial beliefs, values and attitudes are critical for the diffusion of environmental management practices (Rivera and De Leon, 2005). Given the importance of MEC in enterprise development, major enterprises (such as Apple, and Google among others) have adopted the concept to regulate their activities (Riera and Iborra, 2017; MacNeil et al., 2021). Prior studies have attested to how MEC affects the innovation performance of enterprises (Qi et al., 2010; Xue et al., 2019; Song et al., 2021). Xue et al.’s (2019) empirical works on Chinese high-tech enterprises reveal a robust interactive effect of MEC and green innovation on firm performance. Qi et al.’s (2010) studies on the Chinese construction sector conclude that MEC is a dominant driver for the adoption of green practices. Moreover, Lin and Chang (2009) discover a snooping consequence of corporate ecological ethics in the linkage between green relationship learning and GIP. However, the mediating influence of MEC in the CSR activities-GIP link is scant.

Second, GAC describes the enterprise’s ability to procure, integrate, adjust, and exploit eco-friendly knowledge (Chen et al., 2014) for value-creation. The concept acts as a vital element for integrating, transmuting, and applying an outer knowledge source (Cohen and Levinthal, 1990). The GAC permits enterprises to access outer ecological knowledge and then sway inner knowledge to enhance innovation outcomes. However, extant studies on GAC predominantly focus on advanced market firms in which comparatively established concepts have been formed. Contrastingly, emerging market firms are still in their embryonic stage due to considerable variances in structures. Thus, emerging market firms find it difficult to get access to external green knowledge due to weak institutional arrangements, weak research and development centers, and weak infrastructure-if not absent-in relation to the degree of technological innovation (Xie et al., 2018; Boadu et al., 2021a). Therefore, advanced market firms may have the upper hand in the implementation of GAC policies to the detriment of emerging market firms. Previous scholarships have substantiated that GAC can aid enterprises to comprehend the ecological challenges and overwhelmed green torpor (Pacheco et al., 2018), which, in turn, enhances innovation outcomes (Zou et al., 2018; Xue et al., 2019), but the scholarship on the moderation role of GAC on the MEC-GIP linkage is slightly insufficient in the emerging economies.

In addressing these gaps, the present study draws on the theory of planned behavior (TpB), which is one of the most powerful and broadly applied social-psychological models for elucidating human behavior (Han and Stoel, 2017; Yuriev et al., 2020), to develop an integrated model to inspect the impacts of CSR activities on the GIP of the enterprise and consider the mechanism through which MEC and GAC affect the above relationship. First, we assess the correlation between CSR activities and the GIP of the enterprise. Second, we investigate the mediating and direct impact of MEC in the relationship between CSR activities and GIP and GIP, respectively. Finally, we scrutinize the facilitating role of GAC on the MEC-GIP relationship.

In sum, the current study provides new insight on how and when CSR activities enhance the GIP of enterprises, based on the sample data of 358 enterprises from the manufacturing and service sectors in China. First, this paper outlines the research inquiries in the introduction. Second, it puts forward a theoretical model and research hypotheses based on theoretical analysis. Then, it analyses regression based on the research hypothesis and the sample data. The next part deals with the discussion, contribution, limitations, and future trajectory.

## Theory and Hypotheses

### Theory of Planned Behaviour

We adopt the TpB as a theoretical context. TpB is an extension of the theory of reasoned action (TRA) which is based on the concept that the intention of performing a specific behavior by an individual/organization is a consequence of some conscious reasoning (Fishbein and Ajzen, 1975). The concept ponders that working-class comportment is determined by the aim of carrying out certain manners. Thus, the person’s aim is determined by three aspects allied to the preferred upshot of the comportment. These include three independent variables measuring attitudes (i.e., the attitude held en route for the espousal of a specific behavior), subjective norms (i.e., outer social pressure that affects a person to take certain manners), and perceived behavioral control (i.e., a person’s perceived comfort or difficulty of carrying out the precise comportment), that together determine behavioral intention. Authors have applied this theory in different contexts, including predicting persons’ ecological intentions and comportments both inside and outer of enterprises and predicting enterprise’s activities predominantly *via* the behavioral plans of key decision-makers in the organization (Flannery and May, 1994). More especially, authors have used the concept as a theoretical conduit for examining the relationship amongst managers’ characteristics and corporate ecological behavior (Flannery and May, 1994). Admittedly, the proponents of the theory argue that subjective norms and perceived behavioral control play an essential role in shaping a person’s intention to be engaged in environmental matters. In an organizational context, the subjective norms of an individual are shaped by organizational norms (for instance, CSR activities) and peers (leadership) that motivate top executives to engage themselves in different environment-related tasks. Scholars have accentuated the significance of organizational support and leadership for the executives’ environment-specific behavior (Wesselink et al., 2017).

First, while applying TpB to the CSR activities, MEC, GAC, and GIP links, we consider the importance of CSR activities for enterprises in gaining social recognition; and their impact on the behavior and attitude of managers. Given this notion, we, therefore, assume that CSR activities are preceded by implementation intentions. The concept can change some managers’ views on social responsibility from the behavioral attitude depending on the situation facing the enterprise, thus having a positive impact on GIP. The study contends that TpB can aid enterprises to leverage the effect of CSR activities on GIP. Enterprises that learn to utilize CSR activities can create new green processes and product development, which are vital for the competitive advantage. The TpB bids that while the positive green innovation outcome of CSR is not definite, CSR activities are critical to organizational efficacy, competitiveness, and sustainability (Ji and Miao, 2020). Therefore, independent of predictable or unpredicted and desired or unwanted green innovation outcomes in the short and long run, the drive for the primer of CSR activities is to allow green enterprises to achieve their strategic ambitions. Pertinently, the study argues that CSR activities (Ji and Miao, 2020) are an infinite resource that can support enterprises in expounding green innovation consequences. Second, the current scholarship discusses the function of MEC in CSR activities and GIP based on the perspective of TpB. In recent years, authors have recognized that the strength of environmental management pivots on copious internal and external strategic resources (e.g., MEC and GAC) (Xue et al., 2019) to nurture GIP. Admittedly, environmental strategies are predominantly determined by the top executives and form the backdrop for the application of the real environmental undertakings of the enterprise. MEC is the top decision-makers attitude toward environmental issues (Xue et al., 2019). It is suggested that if top decision-makers inherently value the environment, then they will feel that the enterprise should hunt ecological fortification undertakings to promote environmental sustainability. While applying TpB to the MEC, CSR activities, and GIP link, we ponder on the importance of MEC as a critical resource that can play a dispensable function in the relation between CSR activities and GIP. Ecological attitudes of executives coalescing with perceptions of norms for environmental regulation perceived behavioral control, and the past emission reduction activity are taken as predictors of behavioral preferences for source reduction activity in enterprises’ grander green innovation performance. Besides, Executives with greater ecological concern are more probable to take the strategic significance of green activities across the different functional areas in their enterprises. Previous studies support that MEC is beneficial for the concrete eco-innovation performance of enterprises (Qi et al., 2010; Xue et al., 2019; Song et al., 2021). Qi et al.’s (2010) studies on the Chinese construction sector conclude that MEC is a dominant driver for the adoption of green practices.

**FIGURE 1:**
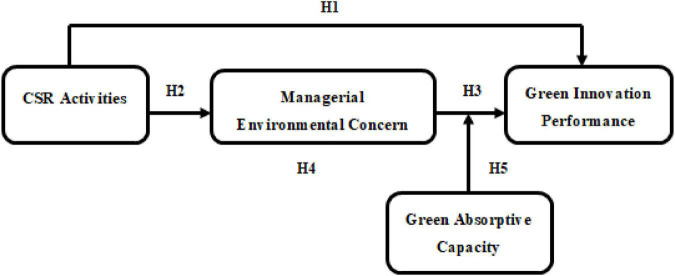
Research model.

Moreover, Lin and Chang (2009) discover a snooping consequence of corporate ecological ethics in the linkage between green relationship learning and GIP. Zhang et al. (2015) studies on the mediating role of senior managers’ environmental concerns in the connection between firms’ energy-saving practices and the external pressures reveal a positive result. We, therefore, argue that enterprises’ green innovation outcomes may be derived from their cognition of MEC. As an environmental concern of the executives is a vital driver for the inclusion of pro-environment elements into their circadian management actions, executives with positive attitudes are ready to lend a hand to the success of proactive environmental practices, which, in turn, lead to the production of behavioral results (i.e., the improvement of GIP). Hence, drawing on the TpB, CSR activities may affect enterprises’ environmental intentions, thereby affecting the perception of MEC, which, in turn, fosters GIP. Third, to advance our comprehension of how MEC mediates the linkage between CSR activities and GIP, the study considers the extent to which the mediation depends on the enterprise’s GAC from the external environment. GAC is the enterprise’s ability to identify the value of external green-related knowledge, incorporate it, transmogrify it into enterprise-embedded green knowledge through integration, conversion, and exploitation capability, and apply it to create value (Cohen and Levinthal, 1990). We contend that with green knowledge top management will be in a better position to supervise product design and development with the least possible adverse effect on the environment. Extant scholarship has evidenced that GAC can empower enterprises to uphold green information gains when gripping and using outwardly diverse green-related knowledge, which aids to get rid of the conceptual precincts of home-grown exploration, and thus has an expanding effect on the GIP results of corporate behaviors (Chen et al., 2015). Thus, the present study contends that GAC can facilitate the impact of enterprises’ MEC to achieve a superior GIP. Figure 1 shows the logical model of the research. The study proposes that CSR activities have an indirect effect on green innovation performance and that managerial environmental concern mediates this link. Besides, the study proposes that green absorptive capacity can moderate the relationship between managerial environmental concerns and green innovation performance.

### Hypotheses Development

#### Corporate Social Responsibility Activities and Green Innovation Performance

Corporate social responsibility describes an enterprise’s voluntary exercise of incorporating environmental, social, and economic concerns into a business’s activities, thus strengthening the affiliation with the enterprise’s interested party (Hang et al., 2022). The concept focuses on several factors that can contribute to addressing the economic, social, and environmental impacts surrounding the organization (Ji and Miao, 2020). Indeed, CSR reflects the excellent behavior of an enterprise in society, and it influences the enterprise’s reputation and social admissibility (Bereskin et al., 2016) which, in turn, enhances overall innovation performance. Under the TpB perspective, enterprise’ CSR practices can be viewed as the proactive and reactive actions of top management in response to unanticipated shocks in the general business environment to gain competitive advantages and grander innovation performance. Major global giants (e.g., Apple, and Google) regard CSR activities as a critical driver for enhancing organizational innovation output (Lin and Lo, 2015). Thus, most enterprises adopt CSR activities as a conduit for continuous innovation to create novel market prospects to emerge and prosper. For example, in cost-effectively subtle enterprises, CSR activities offer significant support to the attainment of ground-breaking consequences echoed in a well-organized application of energy, pollution deterrence, and eco-friendly management (Chen et al., 2006). As regards the relationship between CSR activities and innovation, prior studies have demonstrated divergent results in scrutinizing the correlation between CSR activities and enterprise innovation performance (Gallego-Álvarez et al., 2011; Bocquet et al., 2013; Costa et al., 2015; Reverte et al., 2016; Ueki et al., 2016; Isabel et al., 2017; Wu et al., 2018). Costa et al. (2015) studies conducted on technology exporters confirmed a robust correlation between CSR activities and enterprise exploratory innovation performance. Isabel et al. (2017) discover a robust correlation between CSR activities and innovation outcomes. Wu et al. (2018) discover that CSR can robustly stimulate innovation outcomes with the moderating influence of public visibility. Further, studies conducted by Reverte et al. (2016), who probed the linkage between CSR policies on performance and innovation capacity in Spanish 133 firms reveal a positive association among the variables. Also, studies conducted by Ueki et al. (2016) on Thai trucking enterprises reveal a robust linkage between enterprises’ CSR activities and innovation. At the same time, another stream of studies proved that there is no correlation between CSR activities and corporate innovation performance (Gallego-Álvarez et al., 2011; Bocquet et al., 2013). These findings have created inconsistencies in the CSR and innovation performance literature. Therefore, the presence of inconclusive results suggests a need for further investigation in this area. Considering a firm’s environmental performance, this study contends that CSR activities can support firms to achieve GIP. Through the processes and sequences, firms can execute CSR activities to condense the adverse effects of the enterprises’ activities which, in turn, enhance GIP and competitive advantage.

From the perspective of TpB, CSR activities can play a critical role in identifying an innovative solution to address environmental challenges, which, in turn, improves GIP. Therefore, the current study contends that top-level executive decisions on CSR activities toward environmental matters can effectively change enterprises’ green innovation activities, which, in turn, may enhance the green innovation performance. Accordingly, we state that:

**H1**: CSR activities affect GIP.

#### Corporate Social Responsibility Activities and Managerial Environmental Concern

The significance of CSR activities cannot be over-emphasized in organizational growth, competitiveness, and sustainability (Ji and Miao, 2020). It has become a global phenomenon for enterprises to build their identity and image (Bravo et al., 2012) to enhance their innovation performance and competitive edge (Saeidi et al., 2015). For instance, British Petroleum’s inconsiderateness to social impacts during the enterprise’s Gulf of Mexico oil spill (Lange and Washburn, 2012). Low-carbon demands from customers (Zhu and Geng, 2013). And Boeing’s defectively tested 737 MAX software (Tabuchi and Gelles, 2020). These enterprises have addressed these issues by developing a plan based on CSR activities to circumvent endangering their imminent growth, competitiveness, and financial sustainability (Hemphill, 2005). Thus, a pro-environmental social climate can drive a corporate responsibility to protect the environment and serve the organization. Several enterprises engaging in voluntary environment-related activities target to gratify social expectations (Duarte, 2010). From the perspective of TpB, we contend that top executives are prone to divulge pro-environment penchants when they live in a social community that cares about environmental issues, which further influences decisions made on behalf of the enterprise. Therefore, enterprises need a robust CSR strategy to tackle all social and environmental consequences of their activities to maximize the “shared values” of CSR that reflect firms’ critical stakeholders’ expectations and desires. For instance, enterprises would like to meet customers’ desires for green production approaches to improve their sustainability, particularly in the global market arena (Yin and Ma, 2009). In this vein, CSR activities cannot work in isolation without the business organization’s top executives’ involvement. Admittedly, a vital ingredient for successful CSR activities is MEC which consists of executives or top management teams who can make or unmake enterprise operational activities environmentally friendly (Xue et al., 2019). Flannery and May (1994) revealed a direct link between managers’ characteristics and corporate environmental behavior. Zhang et al. (2015) studies attest to positive support for the relationship between external pressures and managers’ environmental concerns. Zhang et al. (2008) discovered a positive relationship between pressures and environmental management practices.

Drawing on the TpB, we contend that CSR activities can influence managerial environmental concerns to build a well-developed program for the organization and its environs. Fanciful top executives may pay attention to a market penchant, and reluctantly fortify their environmental concerns as a result of the impact of social punter and dealer beliefs and norms. In this context, the current study postulates that CSR activities in Chinese manufacturing and service enterprises can affect MEC. From this we put forward the hypothesis:

**H2**: CSR activities affect MEC.

#### Managerial Environmental Concern and Green Innovation Performance

Linked to the above Hypothesis 2; drawing upon the TpB, environmental concern is one of the most important motives for individual intention about environmental behavior (Wang et al., 2011), which can influence GIP. MEC can be described as the business executives’ commitment or resoluteness to potential ecological menace (Xue et al., 2019). Xue et al. (2019) asserted that the choice between ecological obliteration and ecological protection hinges on decisions by the business’s top management team. For instance, in enterprises facing ecological sturdiness, managerial environmental concerns have a reflective effect on the execution of environmental management activities. Management teams with high commitment and attitude toward environmental sustainability espouse positive practices for plummeting environmental smog, which, in turn, enhances innovation activities. MEC is accentuated as a contributing factor to the enterprise’s innovative behaviors and a fundamental element behind enterprise innovation performance strategies (Qi et al., 2010; Xue et al., 2019; Song et al., 2021). Prior studies have attested how MEC affects the innovation activities of enterprises. Certainly, MEC plays a crucial role in clearing up the germaneness of eco-innovation strategies adoption (Testa et al., 2016; Kumar and Samuel, 2018; Ben Amara and Chen, 2020). The conclusions of the scholarship conducted by Jansson et al. (2010) established that values, beliefs, and norms determine willingness for the espousal of environmental innovation strategies. Scholarship conducted by Qi et al. (2010) using the Chinese construction industry proved a robust association between MEC and green innovation. They attested that MEC fit as the weightiest driver for the espousal of sustainable practices in China. Besides, Lin and Ho’s (2008) scholarship on managerial environmental concerns and green innovation reveals significant impacts among the variables.

Consequently, from the perspective of TpB, MEC can promote enterprises to formulate comprehensive environmental protection policies toward environmental issues, which, in turn, enhance enterprises’ green innovation development. The current study contends that enterprises that incorporate norms and values to draw robust environmental strategies (serving as a critical success factor) from the beginning can drive GIP. Therefore, we suggest that MEC has a positive relationship with GIP. The study states that:

**H3**: MEC has a positive influence on enterprises GIP

#### Mediating Effect of Managerial Environmental Concern

Enterprises with a bundle type of CSR orientation can boost their innovative capability (Costa et al., 2015; Wu et al., 2018). The value of MEC arguably lies in the opportunity to surge environmental management performance, while meeting environmental protection requirements. As such, MEC is perceived not only as an answer to environmental demands but also as an opportunity to boost green innovation capability (Song et al., 2021).

Managerial environmental concern as a concept plays an influential function in building enterprises’ CSR activities. Thus, executives’ commitment affects the speed at which enterprises initiate environmental actions (Song et al., 2021). Such commitment of top executives is momentous to enterprises’ ability to reflect on and solve environmental matters quickly. Notably, a high-level top management team shows proactive concern for environmental innovation (Roxas and Coetzer, 2012; Song et al., 2021). They may strategically increase investment in green technologies (Ji and Miao, 2020). Enterprises with top-notch teams hunt and utilize organizational resources to address environmental practices to enhance innovative output. Conversely, when enterprises’ top management team demonstrates a low level of managerial environmental concerns, they pay little attention to environmental matters (Xue et al., 2019; Song et al., 2021).

The MEC has been presented in different empirical studies primarily as a moderating variable and little empirical evidence, to the best of our knowledge, exists to verify the mediation effect (Lin and Chang, 2009; Qi et al., 2010; Xue et al., 2019; Tan and Zhu, 2022). For instance, Lin and Chang (2009) discover a snooping consequence of corporate ecological ethics in the linkage between green relationship learning and GIP. Tan and Zhu’s (2022) studies proved that ESG ratings significantly promote the quantity and quality of corporate green innovation and are mediated by increasing managers’ environmental awareness. Zhang et al. (2015) studies proved that a senior manager’s environmental concerns play a significant role in the connection between firms’ energy-saving practices and the external pressures such as normative and mimetic pressures. The current study expects to further enrich the literature by exploring the mediation of MEC in the relationship between CSR activities and GIP.

In this context, although CSR activities can have a substantial consequence on GIP, the absence of MEC for ecological matters decreases the robust association amongst the variables due to a high-level top management team support. From the perspective of TpB, we argue that CSR activities emphasize environmental issues to condense the adverse effect of the enterprises’ practices can be well executed through the involvement of high-level top management teams, which, in turn, generate superior GIP. Therefore, the present study postulates that the mediating effect of a high level of MEC exists amongst CSR activities and GIP. Based on the discussion, the study proposes that:

**H4**: Mediating effects of MEC exist amongst CSR activities and GIP.

#### Interactive Effect of Green Absorptive Capacity

Green absorptive capacity acts as a vital element for integrating, transmuting, and applying external green-related knowledge sources (Cohen and Levinthal, 1990). The concept plays a significant role in inter-functional harmonization to benefit enterprises in comprehending the value of external knowledge sources from divergent markets (Naja et al., 2016). The current study argues that enterprise legitimacy hinges on its ability to comprehend external knowledge, adapt it, and apply it to create potential value. Enterprises need the GAC to produce creative and innovative ideas from environmental knowledge (Chen et al., 2015) to build a sustainable competitive advantage. GAC can be classified as high or low. At the highest level of GAC, enterprises can get access to green-related heterogeneous resources and information to enhance their agility and adjustment to institutional pressure. Conversely, in the case of a low-level GAC, enterprises become sedentary in searching for external green-related knowledge and information and create resistance to institutional pressure.

Consequently, the present study uses GAC as a contingency variable on the MEC-GIP link. Extant works have established that GAC supports enterprises’ ecological challenges (Pacheco et al., 2018), which, in turn, enhances innovation outputs (Zou et al., 2018; Xue et al., 2019). Pacheco et al. (2018) have highlighted the importance of green absorptive capacity, which can enhance organizational factors and innovation performance. In the present study, we contend that enterprises with high GAC can work in tandem with managerial environmental concerns stemming from top executives who demonstrate high commitment to environmental opportunities and challenges (Delmas et al., 2011) into GIP. Thus, enterprises’ involvement in external green-related resources and information from new market opportunities in their environmental strategy can influence their overall GIP. Based on the discussions, we propose that:

**H5**:GAC moderates the impacts of MEC on GIP in a positive way. Such that the relationship between MEC and GIP is stronger when GAC is higher.

## Materials and Methods

### Data Collection and Sample

Prior to the questionnaire administration, we conduct a pretest of the questionnaire to ensure its reliability, clarity, and comprehension. First, we engaged a team of academic experts in innovation, international business, and environment fields to assess the draft survey. Second, we engaged 53 MBA students in selected universities in Sichuan province for their views and assessment of the research instrument. The study incorporates all the responses and feedback into the final document for clarity, precision, and ease of use.

We tested the research model empirically by adopting a probability sampling technique (i.e., random) to select 646 enterprises from the manufacturing and service sectors from May 2021 to July 2021. The data collection period lasted for 3-month. Our respondents came from MBA, EMBA, and DBA executives from top universities in southwest China. We use the alumni platform to contact these top executive officers through emails or telephone calls for their consent before mailing them the questionnaire. The study targeted these top executive officers as informants to provide relevant responses to the survey concerning demographic details, corporate social responsibility activities, managerial environmental concerns, green absorptive capacity, and green innovation performance. The study engaged them due to their familiarity with enterprise policies and operational practices. Each questionnaire included a brief introduction highlighting the purpose of the study and ensuring the participants of confidentiality. The respondents were also informed that they could obtain the final results if they returned a completed questionnaire. Follow-up calls and mailings were made 3 weeks after the initial mailing to improve the effective response rate. To protect the respondents’ confidentiality, the finished surveys were sent directly to the researchers. The survey samples were widely distributed throughout China. The study obtained 477 usable responses from the top executive officers, representing 73.84% (477/646). After a thorough examination of the received instruments, the researchers deleted 119 incomplete questionnaires from the sample list and retained 358 surveys, totally representing 55.42% (358/646).

### Variables

The study utilizes a 7-point scale closed-ended survey questionnaire items (Boadu et al., 2021b; Xie et al., 2022a). We designed our scales in English, translated them to Chinese, and back to English to ensure their reliability (Brislin, 1970).

#### Dependent Variable: Green Innovation Performance

We use GIP as our dependent variable. The study adopted four items from Cai and Zhou (2014), Xue et al. (2019) to measure GIP. These include: “Our enterprise processes of producing products or services focus on using clean and recyclable material” “Our enterprise processes of producing products or services condense the consumption of resources, such as water, oil, and electricity” “Our enterprise can successfully condense the emissions of hazardous materials and waste” “Our enterprise has better product quality development or design strategies.”

#### Independent Variable: Corporate Social Responsibility Activities

We utilize CSR activities as our explanatory variable. The concept refers to the social, environmental, ethical, and philanthropic activities executed willingly by the enterprise to meet the anticipations of people (Shum and Yam, 2011). The study adopted the items from Bansal (2005) and Chow and Chen (2012), which consists of four elements to measure CSR activities. These include: “Our enterprise frequently partakes in the social development program to empower citizenry”; “Our enterprise offers a conducive working environment (i.e., safe and healthy) to its workforce”; “Our enterprise respects the human rights of all stakeholders (i.e., workforce, community members, and shareholders) beyond the legal requirements”; “Our enterprise considers the environment as an important component in business activities; Our enterprise offers high-quality products and services to the consuming public.”

#### Mediating Variable: Managerial Environmental Concern

We use MEC to measure the mediating influence in the CSR activities-GIP. The study adapted four items from Xue et al. (2019). These include: “Our enterprise sees environmental innovation as an essential component of environmental management” “Our enterprise regards most environmental innovation as a useful strategy” “Our enterprise sees environmental innovation as an effective environmental management strategy” “Our enterprise sees environmental innovation as a component not necessary to achieve high levels of environmental performance.”

#### Moderating Variable: Green Absorptive Capacity

According to prior scholarships (Xue et al., 2019), we measure GAC with four items from Chen et al. (2014). These include: “Our enterprise can transfer green knowledge across its divisions” “Our enterprise frequently disseminates green knowledge information squarely in all units” “Our enterprise can integrate newly acquired green knowledge into existing green knowledge for developmental activities” “Our enterprise identifies, acquire, and value external green knowledge flows that are critical to its operational activities” “Our enterprise can recognize, acquire, and value external green knowledge which is decisive to its operations.”

#### Control Variables

The study included several control variables that might influence GIP in the analyses. First, we control for enterprise age, which has the potential to influence GIP. Studies have shown that established enterprises tend to perform higher than undeveloped enterprises (Xue et al., 2019). Enterprise age was measured as a natural log of the date of commencement of operation. Second, we control for research and development intensity to establish its influence on GIP. Prior studies have proved that it might be related to innovation performance. We measured research and development expenses as low, medium, and high in relation to main competitors. Third, the type of industry can affect the innovation performance of an enterprise. Due to the differences in resources, production systems, and demand, the innovation ability of enterprises in different categories of business may be disparate (Li et al., 2019). Boadu et al. (2018) contend that enterprises in different categories of business may need different level of knowledge to apply in their environment which can eventually influence enterprises innovative performance. Therefore, we control for industry types (service and non-service), which service is code as (one), and non-service is code as (zero) (Boadu et al., 2018, 2021a) to establish the industry effects on GIP. Fourth, we control for enterprise size, which has the potential to influence GIP. Recent scholarships suggest that large enterprises get access to more resources, which might help establish links with other enterprises. We measured enterprise size as a natural log of the number of workforces (Boadu et al., 2018). Finally, we control for financial subsidy, which has the potential to influence GIP. Extant scholarships have proved that it may be linked with enterprise innovative performance (Boadu et al., 2021a; Tang et al., 2022; Xie et al., 2022a,b). Enterprises were asked to indicate the financial subsidy that they obtain from authorities. This variable takes five values corresponding to the exact subsidy received by a firm (1 = 0–5 M yuan, 2 = 5–20 M yuan, 3 = 20–100 M yuan, 4 = 100–1 B yuan and 5≥1 B yuan; M = million, B = billion). The respondents’ demographics can be seen in Table 1.

**TABLE 1:** Demographic information (*N* = 358).

Feature	Category	Quantity	Percentage
Gender	Male	267	74.58%
	Female	91	25.42%
Age	30 years old and below	93	25.98%
	31–40 years old	173	48.32%
	41–55 years old	71	19.83%
	Over 56 years old	21	5.87%
Education	Undergraduate and below	128	35.75%
	Postgraduate and above	230	64.25%
Tenure	0–2 years	25	6.98%
	2-5 years	67	18.72%
	5–10 years	152	42.46%
	10 years and above	114	31.84%

#### Analytical Technique

The current study divided statistical approaches into two parts. First, the study used SPSS 25.0 and AMOS 22.0 software to assess the reliability, validity, correlation, and regression analysis. Secondly, the study assesses the influence of CSR activities on green innovation performance. The researchers applied a mediation analysis of managerial environmental concern influence in the linkage amongst CSR activities and green innovation performance following the recommendations by Baron and Kenny (1986). Thus, the present study assessed the strength of each predictive variable on the criterion variable by comparing their beta coefficients and the *p*-values. In addition, the study used green absorptive capacity as a moderator on the association between managerial environmental concern and green innovation performance. Finally, the study used the SPSS macro PROCESS to complete the chain moderated mediating effect.

#### Regression Model

Given the theoretical framework designed of the study, our regression formula is as follows:

M1: *MEC* = β_0_ + β_1_ *Age* + β_2_ *R*&*D* + β_3_ *Services* + β_4_ *Size* + β_5_ *Subsidy* + μ

M2: *MEC* = β_0_ + β_1_ *Age* + β_2_ *R*&*D* + β_3_ *Services* + β_4_ *Size* + β_5_ *Subsidy* + β_6_ *CSR* + μ

M3: *GIP* = β_0_ + β_1_ *Age* + β_2_ *R*&*D* + β_3_ *Services* + β_4_ *Size* + β_5_ *Subsidy* + μ

M4: *GIP* = β_0_ + β_1_ *Age* + β_2_ *R*&*D* + β_3_ *Services* + β_4_ *Size* + β_5_ *Subsidy* + β_6_ *CSR* + μ

M5: *GIP* = β_0_ + β_1_ *Age* + β_2_ *R*&*D* + β_3_ *Services* + β_4_ *Size* + β_5_ *Subsidy* + β_6_ *MEC* + μ

M6: *GIP* = β_0_ + β_1_ *Age* + β_2_ *R*&*D* + β_3_ *Services* + β_4_ *Size* + β_5_ *Subsidy* + β_6_ *MEC* + β_7_ *GAC* + μ

M7: *GIP* = β_0_ + β_1_ *Age* + β_2_ *R*&*D* + β_3_ *Services* + β_4_ *Size* + β_5_ *Subsidy* + β_6_ *MEC* + β_7_ *GAC* + β_8_$\bar{M\,E\,C}$*$\bar{G\,A\,C}$ + μ

#### Measurement Testing

We utilize a measurement model to assess the link between latent variables and determinants. Through exploratory factor analysis, we consider the construct uniqueness of the four target variables. Our investigation reveals that all factor loadings are more than the threshold score of 0.60. Besides, we evaluate the reliability and validity test and find that the Cronbach’s values and KMO scores are above the threshold score of 0.70, indicating that each variable is good and satisfies the requirements for the study. We further assess the convergent validity through composite reliability (CR) and average variance extracted (AVE), respectively. From Table 2, the values of CR ranges from 0.8699 to 0.9226 (>0.70, benchmark), whereas the values of AVE ranges from 0.6270 to 0.7488 (>0.5, benchmark). The results signify acceptable convergent validity for all constructs—finally, the study tests for discriminant validity (DV). In testing for DV, studies suggest that the square root value of AVE must be higher than the correlations amongst the construct in the research (Fornell and Larcker, 1981). From Table 3, our results indicate good DV for all constructs (see diagonal values in bold).

**TABLE 2:** Reliability and validity analysis results of each latent variable.

Variables	Item	Factor loading	Cronbach’s alpha	CR	AVE	KMO
Corporate social responsibility activities	CSR1	0.844	0.859	0.8990	0.6407	0.825
	CSR2	0.762				
	CSR3	0.757				
	CSR4	0.797				
	CSR 5	0.838				
Managerial environmental concerns	MEC1	0.889	0.888	0.9226	0.7488	0.820
	MEC2	0.846				
	MEC3	0.837				
	MEC4	0.888				
Green absorptive capacity	GAC1	0.740	0.800	0.8699	0.6270	0.755
	GAC2	0.735				
	GAC3	0.808				
	GAC4	0.876				
Green innovation performance	GIP1	0.904	0.887	0.9216	0.7467	0.806
	GIP2	0.820				
	GIP3	0.818				
	GIP4	0.910				

**TABLE 3:** Mean, SD, and inter-correlations.

Variables	1	2	3	4	5	6	7	8	9
1. Age	1								
2. R&D intensity	−0.023	**1**							
3. Services	−0.012	−0.098	1						
4. Size	−0.065	−0.030	−0.054	**1**					
5. Financial subsidy	−0.001	0.051	−0.031	0.081	**1**				
6. CSR	0.082	0.083	−0.103	0.019	0.087	**(0.800)**			
7. MEC	0.139**	0.141**	−0.172**	0.182**	0.184**	0.315**	**(0.865)**		
8.GAC	0.048	−0.066	0.045	0.091	−0.065	0.037	0.052	**(0.792)**	
9.GIP	0.133*	0.173**	−0.157**	0.100	0.194**	0.211**	0.307**	−0.026	**(0.864)**
Mean	2.011	2.528	0.425	3.028	2.084	3.565	3.590	3.485	3.808
*SD*	0.813	1.229	0.495	1.378	1.213	1.338	1.565	1.327	1.556

N = 358; two-tailed tests. *p < 0.05, **p < 0.01. Inside the brackets is the AVE.

#### Non-response Bias and Common Method Bias

The study conducted a further test on NRB and CMB, respectively. First, to assess whether our dataset is free from NRB, we executed the independent-samples *t*-statistics examination for the means of early and late respondents on four principal demographic variables. We discovered that comparison through t-statistics was not significant. Thus, our dataset is far from NRB, and we can generalize the discoveries to the bigger population (Armstrong and Overton, 1977; Shi et al., 2021). Secondly, we executed CMB, a critical concern in quantitative studies. The study adopted both procedural and statistical techniques to condense any potential CMB problem. In designing our survey instrument, we mixed the order of the predictor, mediator, moderator, and criterion variables. We explained the purpose of the data collection exercise to the respondents and guaranteed that there were no “right” or “wrong” responses for the questionnaire items. Besides, we shielded the identity of the respondents.

Further, we followed Podsakoff et al. (2003) and used Harman’s test of a single factor to assess CMB in the dataset. Our results reveal that the dataset has 7 factors, of which the largest factor contributed 21.92% of the total variance. We, therefore, conclude that CMB is not a severe threat in the dataset, as a single factor contributes <50% of the total variance (Podsakoff et al., 2003).

To further test the adaptability of the validity of the scale construction, this study carried out a confirmatory factor analysis on the sample data. Table 4 reports the results of the overall model of the study: χ^2^/df is <3, RMSEA is <0.08, SRMR is <0.05, and CFI, GFI, TLI, IFI, and NFI are all >0.9. This indicates that the model fit index in this study is ideal level; the validity of the scale structure is good.

**TABLE 4:** Confirmatory factor analysis of the overall model.

Index	χ^2^/df	RMSEA	SRMR	CFI	GFI	TLI	IFI	NFI
Recommended	<3	<0.08	<0.05	>0.9	>0.9	>0.9	>0.9	>0.9
Actual	2.667	0.068	0.0422	0.959	0.934	0.949	0.959	0.937

## Empirical Results

### Descriptive Statistics and Correlation Analysis

Table 3 reports the mean, SD, and correlation of all variables. We find that CSR activities are positively related to MEC and GIP (β = 0.315, *p* < 0.01; β = 0.211, *p* < 0.01), respectively. Besides, MEC is significantly associated with GIP (β = 0.307, *p* < 0.01). We engage regression analysis to run a sequence of tests corresponding to our hypotheses of the study in Table 5. The results show that the VIF value of each model is less than 2, which indicates that the regression results in Table 5 do not have a serious risk of collinearity. Furthermore, the study followed Aiken et al. (1991) and standardized our independent and moderating variables in the regression models.

**TABLE 5:** Regression analysis results.

Variables	MEC: M1 — M2		GIP : M3 — M7				
	M1	M2	M3	M4	M5	M6	M7
Age	0.152 (0.096)**	0.129 (0.093)**	0.142 (0.097)**	0.128 (0.096)**	0.108 (0.096)*	0.109 (0.096)*	0.105 (0.094)*
R&D intensity	0.128 (0.064)*	0.109 (0.062)*	0.157 (0.064)**	0.146 (0.063)**	0.129 (0.063)*	0.128 (0.063)*	0.121 (0.063)*
Services	−0.143 (0.159)**	−0.118 (0.153)*	−0.129 (0.159)*	−0.114 (0.158)*	−0.097 (0.157)	−0.096 (0.158)	−0.089 (0.156)
Size	0.175 (0.057)***	0.171 (0.055)***	0.093 (0.057)	0.09 (0.057)	0.054 (0.057)	0.056 (0.057)	0.062 (0.056)
Financial subsidy	0.159 (0.065)**	0.138 (0.062)**	0.174 (0.065)***	0.161 (0.064)**	0.139 (0.064)**	0.137 (0.064)**	0.136 (0.064)**
CSR		0.268 (0.057)***		0.161 (0.059)**			
MEC					0.221 (0.052)***	0.223 (0.052)***	0.216 (0.052)***
GAC						−0.026 (0.058)	−0.386 (0.142)**
MEC × GAC							0.392 (0.037)**
*R* ^2^	0.112	0.181	0.097	0.120	0.138	0.136	0.159
F	10.044***	14.114***	8.647***	9.103***	10.512***	9.032***	9.434***

*p < 0.05, **p < 0.01, ***p < 0.001; N = 358.

### Test of Hypotheses

#### Main Effect Test

##### Hypothesis Linking Corporate Social Responsibility Activities to Green Innovation Performance

Hypothesis 1 proposes a positive correlation between CSR activities and GIP. As we can see in Model 4, Table 5, the dependent variable is *GIP*, the estimated coefficient on *CSR activities* is positive and statistically significant (β = 0.161, *p* < 0.01, Model 4). This result indicates that CSR activities affect GIP. Thus, Hypothesis 1 is confirmed.

##### Hypothesis Linking Corporate Social Responsibility Activities to Managerial Environmental Concern

Hypothesis 2 predicts that CSR activities affect MEC. The results in Table 5 demonstrate that *CSR activities* had significant positive influence on *MEC* (β = 0.268, *p* < 0.001, Model 2), thus providing support for hypothesis 2.

##### Hypothesis Linking Managerial Environmental Concern to Green Innovation Performance

Hypothesis 3 projects a positive linkage between MEC and GIP. From Table 5, Model 5, our results indicate that *MEC* has a very strong impact on *GIP* (β = 0.221, *p* < 0.001), thus supporting Hypothesis 3.

#### Mediating Effect Test

##### Hypothesis on Mediated Indirect Effect of Managerial Environmental Concern

Hypothesis 4 states that the mediating effects of MEC exist amongst CSR activities and GIP. From Table 5, Model 4, the results show that there is a linear linkage between CSR activities and GIP. Still, the mediating effect of MEC in the linkage amongst CSR activities and GIP could be tested by the traditional methods of Baron and Kenny (1986). Referring to Baron and Kenny’s three criteria recommendations, we assess the mediation of MEC. First, we explore the linkage amongst the independent variable and the criterion variable of the study. Our results in Table 5, Model 4, attest to CSR activities’ influence on GIP (β = 0.161, *p* < 0.01, Model 4, Table 5). Second, we examine how the predictor variable affects the mediator. Our discoveries reveal that CSR activities positively affect MEC (β = 0.268, *p* < 0.001, Model 2, Table 5). Finally, in the regression model, we add the mediator, as we can see, our criterion variable is GIP. The results demonstrate that the estimated coefficient on the mediator variable (i.e., MEC) and a criterion variable (i.e., GIP) are positive and statistically significant (β = 0.221, *p* < 0.001, Model 5, Table 5), respectively. Combining the results of M1–M5, we can predict that the variable (MEC) plays a part in the mediating role among CSR activities and GIP.

In order to further test the robustness of the mediation effect, this paper uses the Process software to conduct Bootstrap analysis (Choose “MODEL 4” in process software, 5,000 samplings, CI = 95%) (see, Table 6). The total effect of CSR activities on GIP is 0.1876, of which the direct effect is 0.1289, accounting for 68.71%; the indirect effect is 0.0587, accounting for 31.29%. At the same time, each model does not contain 0 within the 95% confidence interval, so MEC plays a part in the mediating role among CSR activities and GIP. Hypothesis 4 is supported.

**TABLE 6:** Bootstrap for the mediating effect.

Independent variable	Dependent variable: GIP					
	Type	Size	*S.E*	Bootstrap 95% confidence interval		Ratio
				LLCI	ULCI	
CSR	Total effect	0.1876	0.0586	0.0723	0.3027	100.00%
	Direct effect	0.1289	0.0601	0.0106	0.2471	68.71%
	Indirect effect	0.0587	0.0212	0.0236	0.1062	31.29%

#### Moderating Effect Test

##### Hypothesis on Moderated Effect of Green Absorptive Capacity

We further conduct a moderated effect test to assess our hypothesis 5, which projects that GAC regulates the impacts of MEC on GIP in a positive way. Then, referring to Muller et al. (2005), we add the moderator variable into the regression model. After running the model, we discover that the interactive effects of GAC and MEC relate positively to GIP, such that the relationship between MEC and GIP is stronger when GAC is higher. (β = 0.392, *p* < 0.01, Table 5, Model 7), thus supporting hypothesis 5.

**FIGURE 2:**
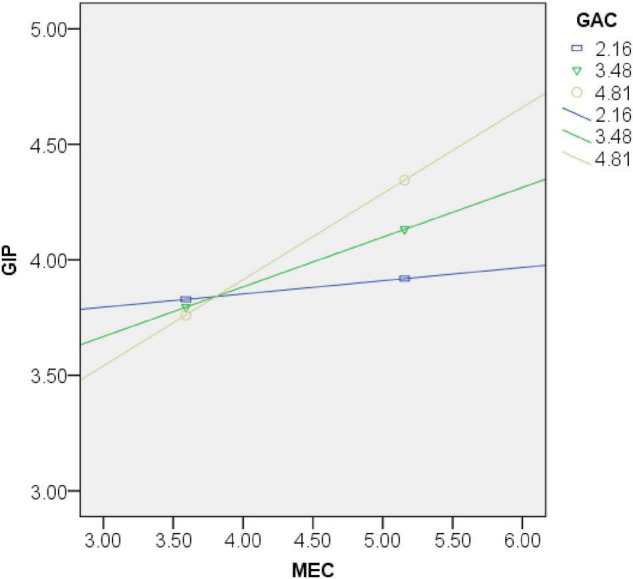
The moderating efffect of green absorptive capacity (GAC).

To analyze the influence of the moderator variables, this paper further uses the process plugin to draw a moderation effect diagram. As displayed in Figure 2, under the condition of high *GAC*, the slope of *MEC* and *GIP* becomes higher. When *MEC* is at the same level, if *GAC* becomes higher, then *GIP* becomes higher. Similarly, if the *GAC* becomes lesser, the *GIP* becomes lesser accordingly. Figure 2 illustrates that the effect of *MEC* on *GIP* is stronger when *GAC* is high (+1 SD). In other words, when *GAC* becomes higher, the *GIP* generated by the same *MEC* becomes higher. Thus, *GAC* positively moderates the relationship between *MEC* and *GIP*, the study finds support for hypothesis 5.

#### Moderated Mediating Effect

##### Moderated Mediating Effect of Green Absorptive Capacity

In order to test the moderated mediating effect which is possibly existed in the framework, we use the PROCESS software to carry out further analysis. We choose M14 (M14 is one of the model numbers in the PROCESS software), set bootstrap samples to 5,000, set the confidence level for confidence intervals to 95%, and then put the independent variables, dependent variables, control variables, intermediate variables, and moderating variable into the corresponding positions for regression analysis, and the report is presented in Table 7.

**TABLE 7:** Moderated mediating effect.

IV	Moderator	CSR → MEC → GIP							
		Conditional indirect effect				Moderated mediating effect			
		Conditional indirect effect	*S.E*	LLCI	ULCI	Coefficient	*S.E*	LLCI	ULCI
CSR	High GAC	0.1065	0.0307	0.0543	0.1752	0.0373	0.0135	0.0152	0.0688
	Low GAC	0.0570	0.0203	0.0231	0.1047				

Results in Table 7 show that GAC has a significant indirect effect on the CSR-MEC-GIP conditional formula, and the confidence interval does not contain 0; at the same time, the moderated mediation effect value is 0.0373, and the confidence interval is [0.0152,0.0688], excluding 0. Therefore, GAC has a significant mediating effect on CSR-MEC-GIP. Finally, we summarize the results of all hypothesis tests of this study in Table 8.

**TABLE 8:** Summary of hypotheses.

Type	Hypotheses	Content	Result
Direct effect	H1	CSR activities have a positive effect on GIP	Verified
Mediation effect	H2	CSR activities have a positive effect on MEC	Verified
	H3	MEC has a positive effect on GIP	Verified
	H4	MEC mediates the positive relationship between CSR activities and GIP	Verified
Moderating effect	H5	GAC moderates the impacts of MEC on GIP in a positive way. Such that the relationship between MEC and GIP is stronger when GAC is higher.	Verified

## Discussion, Implications, and Limitation

Corporate social responsibility activities can play a vital role in enterprise growth, survival, and sustainability. For instance, it can condense conflicts between business and stakeholders such as public sectors, non-governmental organizations, competitors (Blowfield and Frynas, 2005), workforces, consumers, dealers, and the local community (McWilliams et al., 2006), which, in turn, enhance enterprises performance (Wu et al., 2018). However, there are divergent empirical findings on the influence of CSR on enterprise performance in the existing works. Hence, the present study explored the influences of CSR activities on an enterprise’s GIP. Specifically, the study examined the mediating and moderating effects of MEC and GAC on the CSR activities-GIP link. We advance the existing literature in three ways. First, the current scholarship empirically scrutinized the influence of CSR activities on GIP in emerging economies, particularly the Chinese economy. Past research has scrutinized the linkage between CSR activities and innovation performance in various fields. However, prior studies have demonstrated divergent results in scrutinizing the correlation between CSR activities and enterprise innovation outcomes (Gallego-Álvarez et al., 2011; Bocquet et al., 2013; Costa et al., 2015; Reverte et al., 2016; Ueki et al., 2016; Isabel et al., 2017; Wu et al., 2018). From the extant studies, scholars like Isabel et al. (2017) and Wu et al. (2018) assert that CSR activities can robustly stimulate innovation outcomes of enterprises. While, other authors (Gallego-Álvarez et al., 2011; Bocquet et al., 2013) attest that there is no correlation between CSR activities and corporate innovation performance. Our investigations from the regression analysis demonstrate that the combined CSR activities have a robust association with GIP (H1). Our conclusion is somewhat in line with studies conducted by scholars like Isabel et al. (2017) and Wu et al. (2018) that reveal a positive influence factor of CSR activities on innovation outcomes. CSR activities concentrated on economic, social, and environmental issues seem to trigger GIP. Thus, CSR activities serve as a critical driver for enhancing Chinese manufacturing and service enterprises’ GIP. Besides, our findings reveal that CSR activities positively affect MEC (H2). The conclusion is somewhat in line with previous studies (Flannery and May, 1994; Zhang et al., 2008; Zhang et al., 2015). To the best of our knowledge, our research is among the first empirical study to examine the relationship between CSR activities and GIP and CSR activities and MEC. Thus, we provide novel insights into a critical antecedent in cultivating the GIP and MEC of manufacturing and service enterprises in emerging economies. The study further enriches the TpB by investigating important yet understudied CSR activities and their impact on enterprise’ GIP and MEC, respectively.

Second, this study offers new insights into the drivers of GIP. Most of the previous scholarships have scrutinized organizational and institutional drivers of green innovation outcomes (e.g., Rennings, 2000; Demirel and Kesidou, 2011). In recent years, authors have instigated to ponder that green innovation activities are a multifaceted operation that is mainly under a chief executive officer’s decision (Arena et al., 2018). In the current study, we scrutinize the linkage between MEC and GIP in the Chinese manufacturing and service enterprises (H3). Our findings from the regression analysis reveal that the MEC has a robust association with GIP, which is somewhat in line with previous studies that reported the positive influence of MEC on enterprise innovation performance strategies (Lin and Ho, 2008; Jansson et al., 2010; Qi et al., 2010; Testa et al., 2016; Kumar and Samuel, 2018; [Bibr B87][Bibr B7]; Song et al., 2021). The conclusions stress the importance of executives’ commitment to green innovation activities (Liao, 2016; Arena et al., 2018) and contribute to the embryonic research on MEC that can shape an enterprise’s orientation toward the environment and socially responsible activities (Horbach and Jacob, 2018; He and Jiang, 2019).

Third, the study advances the understanding of the mediating effect of the MEC in the CSR activities-GIP link. From the extant works, scholars in the CSR field who have conducted scholarships on the influence of CSR on innovation outcomes have produced inconsistent results (Gallego-Álvarez et al., 2011; Bocquet et al., 2013; Costa et al., 2015; Reverte et al., 2016; Ueki et al., 2016; Isabel et al., 2017; Wu et al., 2018). Our research helps to clarify the inconclusive report in the literature by indicating that the mediating effects of MEC exist amongst CSR activities and GIP (H4). Our theorization is drawn on the TpB and explores to what extent top management teams can influence CSR strategies to promote GIP. Explicitly, we find a positive mediating effect of MEC in the association between CSR activities and GIP, which is somewhat consistent with previous studies that reported the positive mediating influence of MEC in the association between environmental activities and corporate green innovation (Lin and Chang, 2009; Tan and Zhu, 2022). The results demonstrate that the top management team pays significant attention to environmental matters, which, in turn, promote GIP. Therefore, this finding has made a significant step in developing a deeper comprehension of the dynamic of environmental resources and their counterbalancing effects within the innovation literature. The study lays the foundation for how MEC support transfiguring the advantage of CSR activities into GIP. Thus, our conclusions augment the evolving body of experiential clues supporting the legitimacy of TpB at a CSR, MEC, and green innovation levels of analysis.

Fourth, the study investigates the two-way effect of GAC and MEC on GIP (H5). The conclusions divulge the vital role of GAC in regulating the impacts of MEC on GIP. Specifically, the interaction of high GAC and MEC can lead to the more significant effective execution of ecological strategies, which improve the GIP. Thus, the study contends that the variable (GAC) can help enterprises break through the barriers to acquiring and absorbing green external knowledge from inter-intra network channels to strengthen MEC, which, in turn, enhances GIP. Thus, the study is among the first to examine the combined effects of these variables. The conclusions from the regression analysis show a positive moderating effect of GAC on the association between MEC and GIP, and the moderated mediation effect of “CSR activities-MEC-GIP,” expands the application boundaries of GAC in the fields of CSR and GIP. Our investigation demonstrates that GAC acts as a vital element for integrating, transmuting, and applying external green-related knowledge sources (Cohen and Levinthal, 1990) to create value. It supports enterprises to comprehend the environmental challenges and overwhelmed green torpor (Pacheco et al., 2018), which, in turn, enhances innovation outcomes (Zou et al., 2018; Xue et al., 2019). Our research thus extends the application of the absorptive capacity theory to CSR literature and shows how GAC sets the boundary condition of the impact of MEC on GIP.

The present study has vital implications for business executives and policymakers. First, our study offers an essential insight into CSR activities by enterprises in emerging economies, particularly in China. Our discoveries suggest that CSR activities appear to increase GIP. Therefore, it is worth for other enterprises’ executives’ pay attention and invests adequately in CSR activities to enhance green innovation strategies. More especially, executives can undergo regular training to progressively change their earlier prejudiced view that investment in CSR activities is merely increasing costs. This will in turn assist in moving beyond the negative view of the cost theory in favor of paying attention to the function of CSR activities in enhancing enterprises MEC and GIP, respectively. Second, achieving GIP requires a shift in management’s view of CSR activities and a renewed concentration on management incentives. This study stress CSR activities in terms of the diffusion mechanism to improve executive environmental protection awareness, and it is also an essential element that top executive cannot ignore when making green innovation decisions. Top executive awareness is the epitome of enterprise strategy. Therefore, it is worth that executives need to understand the MEC clearly. Indeed, they need to take a long-term perspective of MEC, as benefits accruing from MEC can be low or high. The current paper demonstrates that enterprises that emphasize deep MEC are more likely to record better GIP, which, in turn, leads to a sustainable competitive edge in the market environment. In other words, the MEC can influence the efficient use of CSR activities, thereby boosting the efficiency of GIP. Third, our findings show that GAC positively impacts the correlation between CSR activities and GIP. Given the nature of the outcome, the study suggests that executives must apply high GAC acquire through the external source that embraces environmental know-how to augment the internal source to enhance environmental strategy, which, in turn, can affect changes in the overall GIP. Executives should nurture knowledge flows and unceasingly monitor vicissitudes in environmental regulations so that they can detect the market prospects and satisfy social norms. Fourth, policymakers should continue to enact laws and policies on environmental issues that may affect its industrialization drive. Finally, policymakers must create an enabling environment full of strong institutions and regulations to deal with environmental offenders.

The current investigation is without a limitation, which future research should endeavor to address. First, the current scholarship only discusses the mechanism and boundary conditions of CSR activities and their effect on enterprises’ green innovation performance from the perspective of the theory of planned behavior. In the future, researchers should further explore corresponding mechanisms and boundary conditions from other perspectives, and pay attention to other boundary conditions. The impact of CSR activities on the enterprises’ pro-land behavior (Xie et al., 2022b) could also be explored, to enhance the stability of results. Second, this study model only focused on and analyzed the moderating effects of GAC. In the future, two moderating variables (such as corporate governance and organizational culture) can be studied simultaneously, in both their positive and negative aspects, or with one variable negative and the other positive, which should have an impact on the model as a whole. Third, the study adopts a cross-sectional approach; we suggest that future researchers should focus on longitudinal design to investigate the relationship between the variables to optimize these measurement indicators and obtain broader conclusions. Fourth, in the existing scholarship, MEC mediates the association between CSR activities and green innovation performance. So, more scholarships can analyze this linkage with the presence of mediating variables such as green knowledge sharing or entrepreneurial opportunities (Boadu et al., 2021a; Tang et al., 2022) to further advance our understanding of the issue. Furthermore, the sample of the current scholarship is limited to particular sectors (i.e., manufacturing and service) of the Chinese economy; the validity of the model may be affected by social and cultural factors. Thus, it limits the generalizability of the study results. Given the importance of the issue, future research should examine it further in contexts beyond the manufacturing and service sectors. Besides, future research should consider collecting samples from other countries to re-test the research model.

## Conclusion

In recent years, the concept of CSR activities reached a concrete and prime position in the discussion of a critical driving element in accomplishing firms’ strategic vision, survival, and competitiveness in today’s turbulent market environment. Major enterprises in both industrialized and emerging markets have acknowledged the concept and integrated it to distinguish their corporate activities for a competitive edge. Existing scholarships have focused mainly on the effects of CSR activities on firm innovation capability and performance. However, there is insufficient research on the effects of CSR activities on GIP in emerging economies and, more significantly, the mediating and moderating factors that facilitate this link. This research relies on a sample of 358 enterprises from the manufacturing and service sectors in China; and uses regression and bootstrap to test the hypotheses proposed. The empirical results demonstrate that (1) CSR activities affect GIP; (2) CSR activities influence MEC; (3) MEC mediated the linkage between CSR activities and GIP; (4) MEC has a powerful influence on GIP; (5) GAC moderates the effect of MEC on GIP. This study further enriches the theoretical and empirical research achievements in CSR activities and GIP of enterprises with the mediating and moderating impact of MEC and GAC. However, in emerging economies, enterprises’ applications of MEC and GAC are still in their infancy due to significant differences in systems. Policymakers should not only strengthen the application of command and control instruments but also create an avenue to assist enterprises in the acquisition of green knowledge from the external environment. They should also accelerate incentive−based methods such as tax holidays to enterprises that adopt MEC toward GIP.

## Data Availability Statement

The raw data supporting the conclusions of this article will be made available by the authors, without undue reservation.

## Author Contributions

XM, FB, YL, ZC, and AO contributed to conception and design of the study, analyzed the data, and wrote the initial draft of the manuscript and putting forward the main propositions. XM and FB collected the data. FB and AO were responsible for reviewing and editing the manuscript. All authors contributed to manuscript revision, and read and approved the submitted version.

## Conflict of Interest

The authors declare that the research was conducted in the absence of any commercial or financial relationships that could be construed as a potential conflict of interest.

## Publisher’s Note

All claims expressed in this article are solely those of the authors and do not necessarily represent those of their affiliated organizations, or those of the publisher, the editors and the reviewers. Any product that may be evaluated in this article, or claim that may be made by its manufacturer, is not guaranteed or endorsed by the publisher.
